# Refrigeration Performance and Entropy Generation Analysis for Reciprocating Magnetic Refrigerator with Gd Plates

**DOI:** 10.3390/e20060427

**Published:** 2018-06-01

**Authors:** Yonghua You, Zhongda Wu, Yong Yang, Jie Yu, Dong Zhang, Zhuang Zhang

**Affiliations:** 1 State Key Laboratory of Refractories and Metallurgy, Wuhan University of Science and Technology, Wuhan 430081, China; 2 National-Provincial Joint Engineering Research Center of High Temperature Materials and Lining Technology, Wuhan University of Science and Technology, Wuhan 430081, China; 3 School of Material and Metallurgy, Wuhan University of Science and Technology, Wuhan 430081, China

**Keywords:** reciprocating magnetic refrigerator, Gd plate, refrigeration performance, entropy generation, 2D numerical simulation

## Abstract

In the current work, a novel 2D numerical model of stationary grids was developed for reciprocating magnetic refrigerators, with Gd plates, in which the magneto-caloric properties, derived from the Weiss molecular field theory, were adopted for the built-in energy source of the magneto-caloric effect. The numerical simulation was conducted under the conditions of different structural and operational parameters, and the effects of the relative fluid displacement (φ) on the specific refrigeration capacity (qref) and the Coefficient of Performance (COP) were obtained. Besides the variations of entropy, the generation rate and number were studied and the contours of the local entropy generation rate are presented for discussion. From the current work, it is found that with an increase in φ, both the qref and COP followed the convex variation trend, while the entropy generation number (Ns) varied concavely. As for the current cases, the maximal qref and COP were equal to 151.2 kW/m^3^ and 9.11, respectively, while the lowest Ns was the value of 2.4 × 10^−4^ K^−1^. However, the optimal φ for the largest qref and COP, and for the lowest Ns, were inconsistent, thus, some compromises need be made in the optimization of magnetic refrigerators.

## 1. Introduction

Magnetic refrigeration near room temperature is a potential alternative to the traditional vapor compression counterpart [1,2,3]. An active magnetic regenerator (AMR), with the skeleton being manufactured by solid magneto-caloric materials (MCMs), is the key component of advanced room-temperature magnetic refrigerators. A typical magnetic refrigeration cycle consists of the four processes of magnetization, including heat rejection, demagnetization, and heat absorption, and its performance depends on a variety of factors. In addition to developing new MCMs [4,5], scholars and scientists have made great efforts to improve magnetic refrigerator performance from various aspects, such as efficient magnets [6,7,8], advanced refrigeration cycles [9,10], heat transfer enhancement [11,12,13,14,15], smart mechanical design [16,17,18,19,20], and optimal control [21,22]. On the other hand, in addition to building prototypes for experiments [23,24], various 1D and 2D numerical models, based on solving the energy conservation equations of fluid and solid MCMs, have been developed for the research of magnetic refrigeration [2,10,11,12,13,14,15,25,26], and Nielsen et al. have made a review on the AMR modeling [27]. In these models the magnetic refrigeration fundamental was studied [10,12] and the effects of structural and operational parameters on performance aspects, such as temperature span, refrigeration capacity, and Coefficient of Performance (COP), were studied for optimization [28,29,30]. With the assumption of local thermal equilibrium between the fluid and solid phases, Teyber et al. developed a semi-analytic AMR model with two-layer MCMs [31], which could be extended for the optimization of AMRs with multi-layer MCMs.

As is well known, entropy generation takes place in an irreversible process [32], and the minimal entropy generation principle can be applied for the optimal design of various thermal equipment [33,34,35]. With the assumption of trivial fluid thermal capacity, Rowe and Barclay proposed an expression of temperature for optimal magneto-caloric effects, from the aspect of entropy generation minimization [36]. Li et al. [37] analyzed the irreversible losses in magnetic refrigerators and performed geometrical optimization for an AMR with Gd particles. Lei et al. [38] and Trevizoli et al. [39] conducted parameter optimizations for AMRs, with different geometries, based on the entropy generation minimization. 

Compared with their counterpart of packed particles, the AMRs with MCM plates have the advantage of smaller flow resistance. A magnetic refrigeration apparatus, with an AMR of Gd plates, was built in the Risø DTU (Technical University of Denmark) [19] and a 2D numerical model of sliding grids was developed for it [26]. Both experiments and the numerical simulation of Risø DTU demonstrated that the refrigerator with MCM plates could generate a notable temperature span. To further improve the refrigerator performance of Risø DTU, thinner Gd plates, together with a smaller pitch, were adopted in our previous Computational Fluid Dynamics (CFD) simulation of a reciprocating refrigerator and the maximal temperature span was greatly improved. It is noted that the Navier–Stokes equations need be solved hundreds of thousands of times in the commercial CFD simulation of magnetic refrigerators and, thus, the computation is very time-consuming.

In the current investigation, a novel 2D numerical model based on stationary grids will be developed with Matlab for the reciprocating magnetic refrigerators with MCM plates. With the 2D model, numerical simulations will be performed to study the effects of structural and operational parameters on the refrigeration capacity and the COP of magnetic refrigerators. Moreover, the variations of the entropy generation rate and number with structural and operational parameters will be studied, and the contours of the local entropy generation rate will be presented for discussion.

## 2. Physical Model

**Figure 1:**
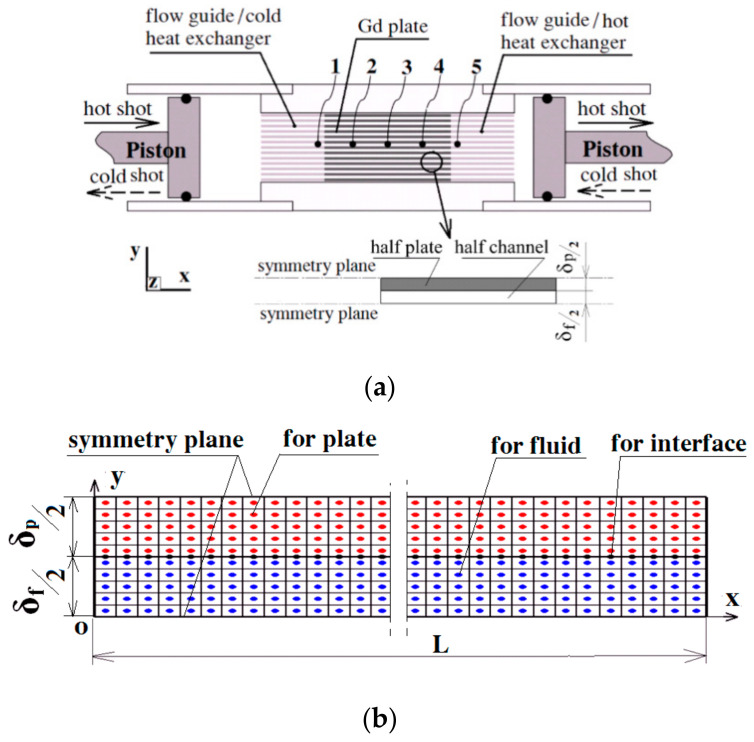
Reciprocating magnetic refrigeration apparatus manufactured with Gd plates [19] and computation grids of the unit structure. (**a**) Magnetic refrigeration apparatus; and (**b**) the computation domain and grids generation.

Figure 1a depicts the reciprocating magnetic refrigeration apparatus of Gd plates studied by the Risø DTU [19,26]. In their apparatus, the thickness (δ_p_) and streamwise length (L) of the Gd plates were 40 mm of 0.9 mm, respectively, and the channel width, or the gap, between two adjacent plates (δ_f_) was 0.8 mm. Five thermocouples were arranged to measure the fluid temperatures and the temperature span (∆T_HC_) was determined by the subtraction of the 5th and 1st thermocouples under the no load condition. During the hot (or cold) blow, the two pistons on the two ends of the apparatus moved synchronously towards the right (or the left). The magnetic field (μ_0_H) at the center of the pole gap was ~1.0 T, while the average field in the “out of field” position was ~0.16 T due to the stray field of the electromagnet [19].

The current investigation is based on the above refrigeration apparatus. To enhance refrigeration performance, the magnetic field at the pole center had an intensity of 1.4 T and the AMR is demagnetized, with the field at approximately 0 T. Two thicknesses were adopted for the Gd plates, i.e., δ_p_ = 0.4 or 0.8 mm, while the channel width (δ_f_) was kept constant at 0.4 mm. Furthermore, the cycling period (τ) was 0.5, 1 or 4 s. Deionized water acted as the working fluid, and the hot and cold reservoirs were at the temperatures of T_H_ (=300 K) and T_C_ (=286 K), resulting in a temperature span of 14 K. The piston stroke is expressed by the relative fluid displacement (φ), which is calculated by:

(1) $$\varphi =\frac{u_{p}{\tau (\delta }_{p}{+\delta }_{f})}{{4\delta }_{f}L}$$

Here, u_p_ and τ/4 refer to the piston velocity and the duration time of the hot blow, respectively.

## 3. Numerical Model and Computation Scheme

### 3.1. Governing Equations

During the magnetic refrigeration cycle, the solid MCMs were subjected to a magnetic field of varied intensity. With the consideration of the effect of the magnetic power, the refrigeration process could be expressed by the following fluid and solid energy conservation equations [26,27,35]:

(2) $$\rho_{f}c_{f}\frac{\partial T_{f}}{\partial t}+\rho_{f}c_{f}(u\cdot \nabla )T_{f}=\nabla \cdot (k_{f}\nabla T_{f}+\bar{\bar{\tau }}\cdot u)$$

(3) $$\frac{\partial (\rho_{s}c_{s}T_{s})}{\partial t}=\nabla \cdot {(k}_{s}\nabla T_{s}{)+q}_{\mathrm{MCE}}$$

where T and t refer to the temperature and time, respectively, while the subscripts f and s represent the fluid and the solid, respectively. $\bar{\bar{\tau }}$ represents the viscous stress tensor and: $\bar{\bar{\tau }}=\mu (\nabla u+\nabla u^{T}-\frac{2}{3}\nabla \cdot \mathrm{uI})$.

As for the refrigerators manufactured by the MCM plates with a small pitch (see Figure 1a), the fluid flow between adjacent plates could be assumed to be 2D laminar and fully-developed, which indicates that the y-component velocity in a typical small channel equals zero, while the x-component counterpart is calculated by [26]:

(4) $$u_{x}=u_{p}\cdot (\delta_{p}+\delta_{f})\cdot (\frac{3}{2\delta_{f}}-\frac{6y^{2}}{{\delta_{f}}^{3}})$$

The q_MCE_ of Equation (3) represents the energy source caused by the magneto-caloric effect of the MCMs. With the application of the thermodynamics equation of Maxwell, the energy source could be expressed by [11,26,27]:

(5) $$q_{\mathrm{MCE}}=-T(\frac{\partial s}{\partial \mu_{0}H}{)}_{T}\cdot \frac{\partial \mu_{0}H}{\partial t}$$

Here, the specific entropy (s) of the MCMs is calculated with the Weiss molecular field theory [25,40,41].

Lastly, the fluid and solid temperature fields in Equations (2) and (3) were coupled with the conservation of heat flux through the interface, i.e.,:

(6) $$k_{f}{\frac{\partial T_{f}}{\partial n}|}_{w^{+}}{=k}_{s}{\frac{\partial T_{s}}{\partial n}|}_{w^{-}}$$

where n represents the normal direction against the fluid-solid interface.

### 3.2. Computation Domain, Mesh Generation, and Solution Scheme

To minimize the computation load, the 2D unit structure, consisting of half a typical channel and half an MCM plate, was adopted as the spatial computation domain, and uniform grids were adopted for mesh generation, as depicted in Figure 1b. The spatial derivatives in Equations (2) and (3) were discretized with the central-difference scheme, while the scheme of fully-implicit forward differences was adopted for the unsteady terms. The software package of Matlab was adopted in the current computation. The solution scheme is similar to the scheme in You et al. [11], i.e., the discretized algebraic equations were solved with the sparse decomposition algorithm, and the whole solution procedure consisted of two iteration loops: The external one is for the periodical steady running, while the temperature fields of an entire cycle were obtained with the internal loop. The independence of the solution, on both spatial and temporal grids, were checked. With the compromise between the computation load and precision, the final computation of a typical case, with 0.8 mm plates, used 10 × 60 spatial grids together with 2400 time steps per cycle, and the iterative convergence criterion was set as 1.0 × 10^−6^.

### 3.3. Model Validation

The current specific entropy, specific heat, and adiabatic temperature increment of Gd, obtained by the Weiss molecular field theory, were compared with the experimental and numerical counterparts in the literature [19,26], and good agreement is observed among them.

The magnetic refrigerator in Ref. [19] was computed with the current 2D model for validation. The predicted maximal temperature spans (∆T_max_) at various relative fluid displacements, obtained by the interpolations at zero refrigeration capacity, were consistent with their experimental counterparts in the literature. As the Weiss molecular field theory could overrate magnetic entropy variation and underrate specific heat near the Curie temperature [2], a mean overestimation of ~2.17 K was observed in the current model validation.

## 4. Numerical Results and Discussions

### 4.1. Calculations of Refrigeration Performance and Entropy Generation

#### 4.1.1. Refrigeration Capacity and Coefficient of Performance

The refrigeration capacity and heat rejection per AMR volume, expressed by q_ref_ and q_rej_, respectively, are calculated by [11]:

(7) $$q_{ref}=\frac{2}{(\delta_{f}+\delta_{p})L\tau }\int_{0}^{\tau }\int_{0}^{0.5\delta_{f}}\rho_{f}c_{f}u_{x}\cdot (T_{C}-T_{f,x=0})\mathrm{dydt}$$

(8) $$q_{rej}=\frac{2}{(\delta_{f}+\delta_{p})L\tau }\int_{0}^{\tau }\int_{0}^{0.5\delta_{f}}\rho_{f}c_{f}u_{x}\cdot (T_{f,x=L}-T_{H})\mathrm{dydt}$$

The pressure drops of the hot and cold blows were calculated by empirical correlation. As the working fluid flowed into the smooth straight channel with a limited velocity, the pumping power consumption was trivial compared with the heat transfer rate and, thus, the Coefficient of Performance (COP), i.e., the ratio of the refrigeration capacity against the total power consumption could be calculated by:

(9) $$COP=\frac{q_{ref}}{q_{rej}-q_{ref}}$$

#### 4.1.2. Specific Entropy Generation Rate and Entropy Generation Number

As is well known, the entropy generation due to heat conduction can be calculated by $s_{g,\Delta T}=k{\left|\nabla T\right|}^{2}/T^{2}$, thus, the entropy generation rate per AMR volume induced by the heat transfer in the fluid and solid MCMs, respectively, expressed by $S_{g,\Delta T}^{f}$ and $S_{g,\Delta T}^{s}$, could be obtained by the integration of the local entropy generation rate over their corresponding computation domains. For example, the specific entropy generation rate of the fluid heat transfer is calculated by:

(10) $$S_{g,\Delta T}^{f}=\frac{2}{(\delta_{f}+\delta_{f})L\tau }\int_{0}^{\tau }\int_{0}^{L}\int_{0}^{\frac{\delta_{f}}{2}}\frac{k_{f}{\left|\nabla T\right|}^{2}}{T^{2}}\mathrm{dydx}dt$$

Similarly, the local viscous entropy generation rate could be calculated by $s_{g,\Delta p}=\frac{\mu }{T}\frac{\partial u_{i}}{\partial x_{j}}(\frac{\partial u_{i}}{\partial x_{j}}+\frac{\partial u_{j}}{\partial x_{i}})$, where the tensors, u and x, have subscripts, i and j, and the viscous entropy generation rate per AMR volume could be obtained by:

(11) $$S_{g,\Delta p}^{f}=\frac{2}{(\delta_{f}+\delta_{p})L\tau }\int_{0}^{\tau }\int_{0}^{L}\int_{0}^{\frac{\delta_{f}}{2}}\frac{\mu }{T}\frac{\partial u_{i}}{\partial x_{j}}(\frac{\partial u_{i}}{\partial x_{j}}+\frac{\partial u_{j}}{\partial x_{i}})dydxdt$$

With the assumption that the irreversible losses of the magnetization and demagnetization were trivial, the specific entropy generation rate of the total AMR is equal to the sum of the fluid and solid counterparts, i.e., $S_{g}^{\mathrm{AMR}}=S_{g,\Delta p}^{f}+S_{g,\Delta T}^{f}+S_{g,\Delta T}^{s}$. On the other hand, the total entropy generation rate could be calculated by the entropy balance equation, i.e.,:

(12) $$S_{g}^{\mathrm{AMR}}=\frac{2}{(\delta_{f}+\delta_{f})L\tau }\int_{0}^{\tau }\int_{0}^{0.5\delta_{f}}\rho_{f}u_{x}(s_{x=L}^{f}-s_{x=0}^{f})dyd\tau$$

For a more rational comparison among different cases, the entropy generation number, normalized by refrigeration capacity, i.e., Equation (13), was adopted in the current study.

(13) $$Ns=S_{g}^{\mathrm{AMR}}/q_{\mathrm{ref}}$$

### 4.2. Variations of Refrigeration Performance with Relative Fluid Displacement

**Figure 2:**
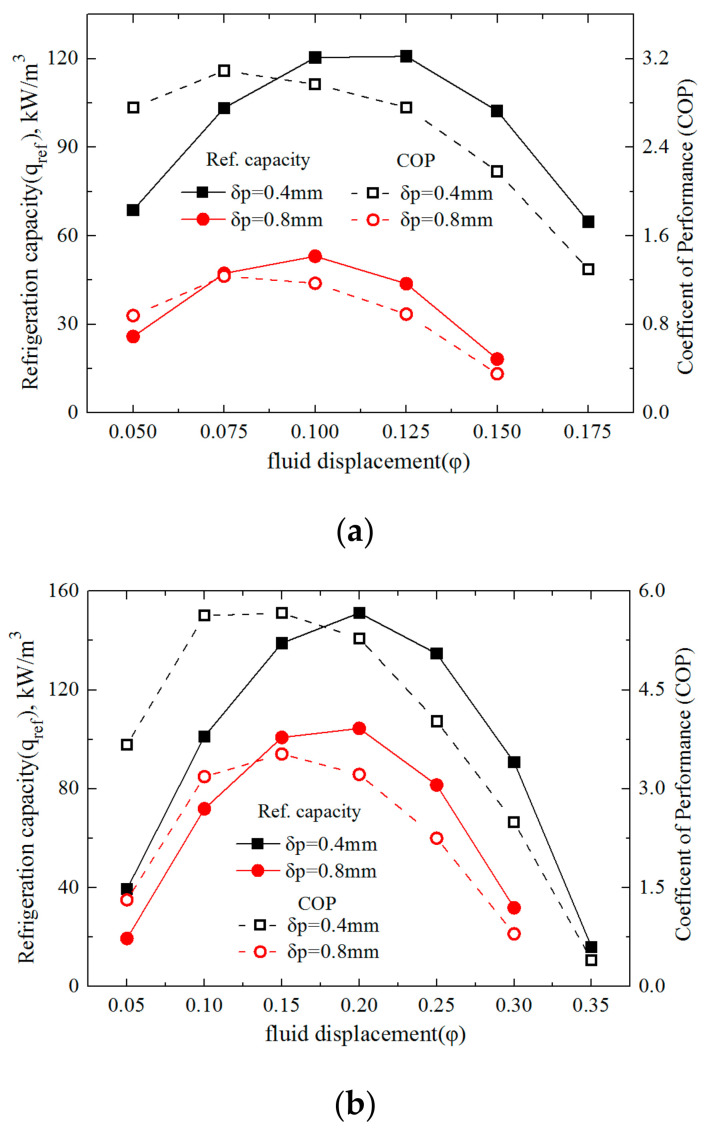

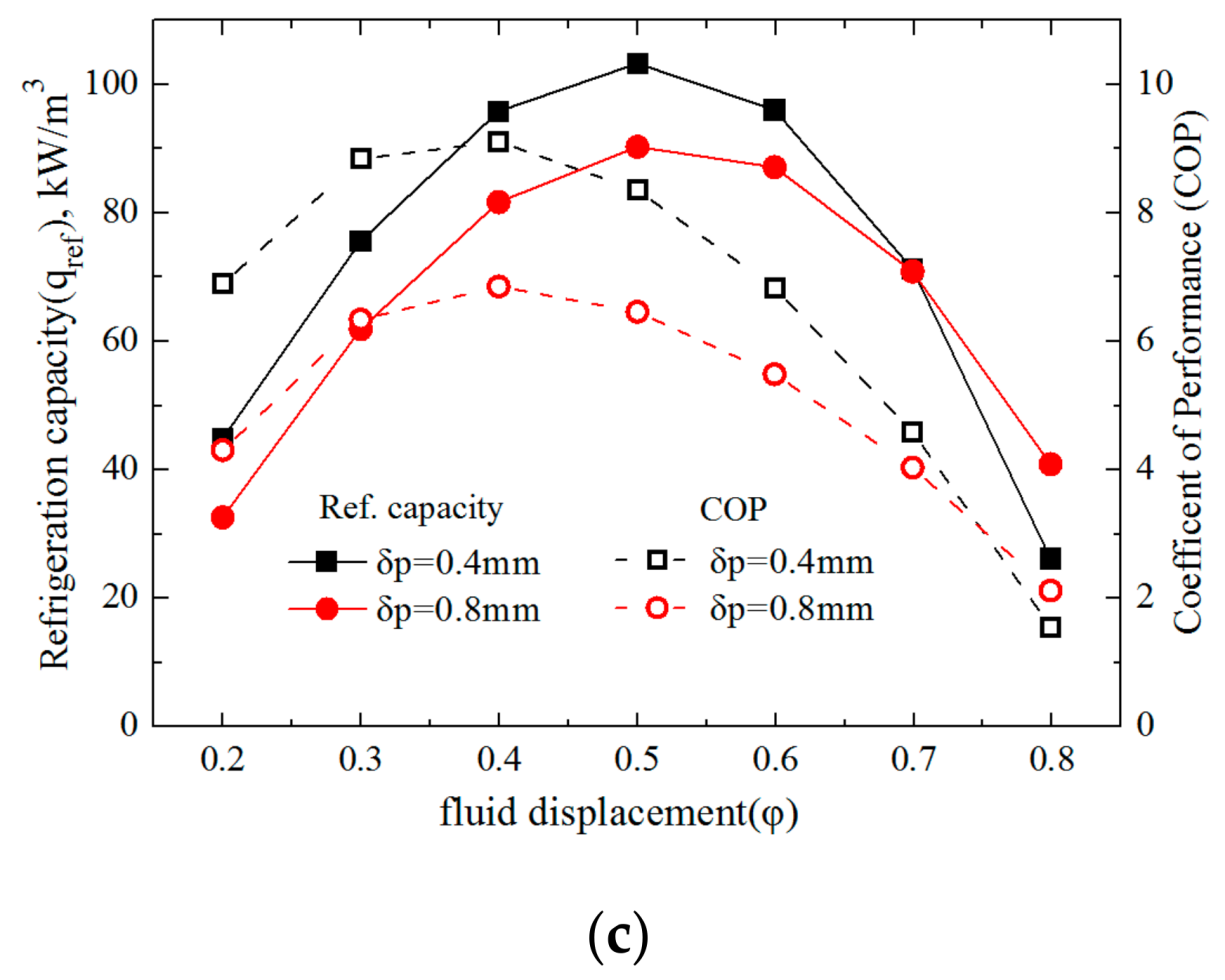
Variations of the specific refrigeration capacity and the Coefficient of Performance (COP) with relative fluid displacement under different cycling periods (τ). (**a**) τ = 0.5 s; (**b**) τ = 1 s; (**c**) τ = 4 s.

The variations of the specific refrigeration capacity and the Coefficient of Performance with relative fluid displacement under the cycling periods of 0.5, 1 and 4 s, respectively, are presented in Figure 2a–c, with the double vertical coordinates where the two plates’ thickness (δ_p_ = 0.4, 0.8 mm) were adopted in the AMRs.

#### 4.2.1. Variations of Specific Refrigeration Capacity

From the left vertical axes of Figure 2a–c, it is observed that the two AMRs both generated the specific refrigeration capacity (q_ref’_) to vary convexly with an increment of the relative fluid displacement (φ) for all the cycling periods (τ), which indicates that an optimal φ could be taken for the maximal q_ref’_. Moreover, the optimal φ increased with an increment of τ, and a moderate τ facilitated a larger peak of the q_ref’_. Specifically, under the conditions that τ equals 0.5, 1 and 4 s, the AMR, manufactured by the Gd plates of δ_p_ = 0.4 mm, had the optimal φ of 0.125, 0.2 and 0.5, and the corresponding peak of the q_ref’_ were equal to 120.8, 151.2 and 103.3 kW/m^3^, respectively. Moreover, it is found that the thinner Gd plates generated a larger q_ref’_, especially at a smaller τ. Specifically, when τ was 0.5, 1 and 4 s, the peak of the q_ref_ generated by the AMR, with Gd plates of δ_p_ = 0.8 mm, was about 43.9%, 69.1%, and 87.4% of the counterparts of the Gd plates of δ_p_ = 0.4 mm, respectively.

#### 4.2.2. Variations of the Coefficient of Performance

The right vertical axes in Figure 2a–c depict the variations of the Coefficient of Performance (COP) with relative fluid displacement (φ) under the cycling periods (τ) of 0.5, 1 and 4 s, respectively. Similar to the specific refrigeration capacity, the COP of the AMR follows the convex variation trend, with φ for all the τ. However, the largest peak of the COP was generated at the greatest τ. Furthermore, the AMR with thicker plates was found to go against a better COP, especially under the condition of a small τ. As for the AMR manufactured by the Gd plates of δ_p_ = 0.4 mm, when the τ of 0.5, 1 and 4 s were adopted, the peak of the COP was 3.09, 5.67 and 9.11, respectively, while their optimal φ were equal to 0.075, 0.15 and 0.4, respectively. It is clear from Figure 2a–c that the optimal φ, corresponding to the maximal COP, was smaller than the counterpart of the maximal q_ref_.

### 4.3. Variations of Entropy Generation with Relative Fluid Displacement

#### 4.3.1. Variations of Specific Entropy Generation Rates

**Figure 3:**
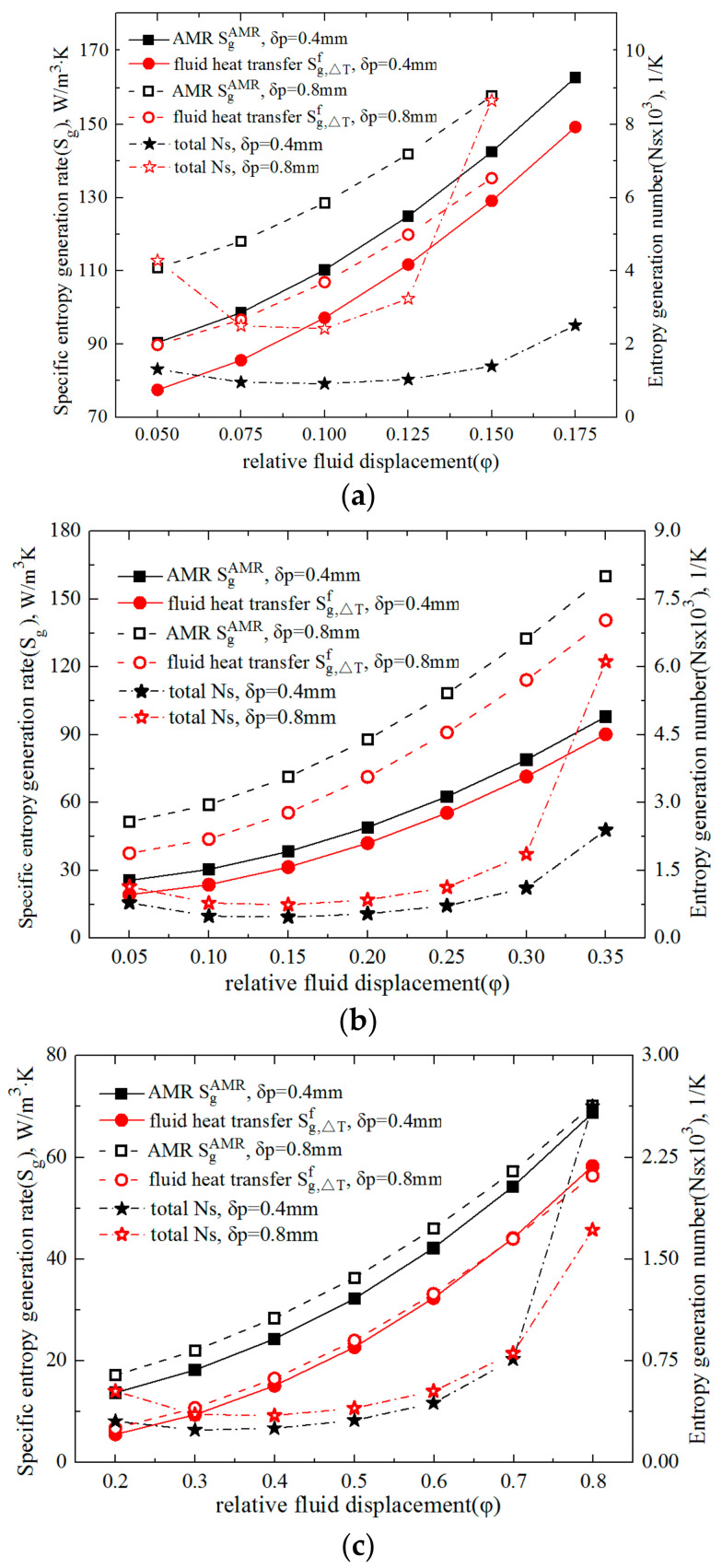
Variations of the specific entropy generation rate of fluid heat transfer and the total active magnetic regenerator (AMR) ($S_{g,\Delta T}^{f},\;S_{g}^{\mathrm{AMR}}$), along with that of the AMR entropy generation number, with relative fluid displacement under different cycling periods (τ). (**a**) τ = 0.5 s; (**b**) τ = 1 s; (**c**) τ = 4 s.

**Figure 4:**
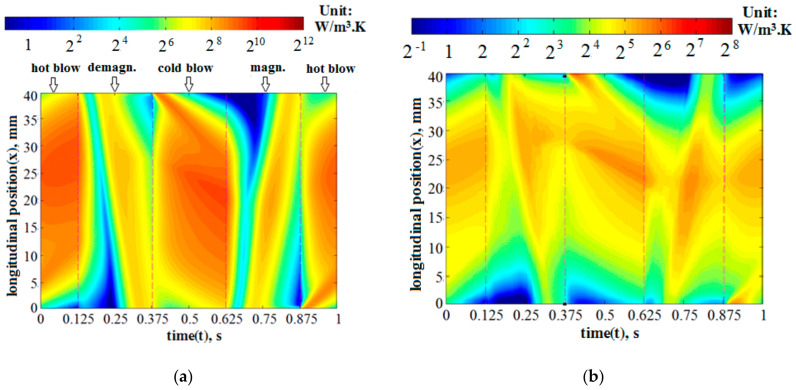
Contours of local entropy generation rates induced by heat transfer, using the log values based on 2, for the cases with the fluid displacement equal to 0.15, where an entire cycling period is depicted. (**a**) Mean fluid entropy generation rate; and (**b**) mean solid entropy generation rate.

**Figure 5:**
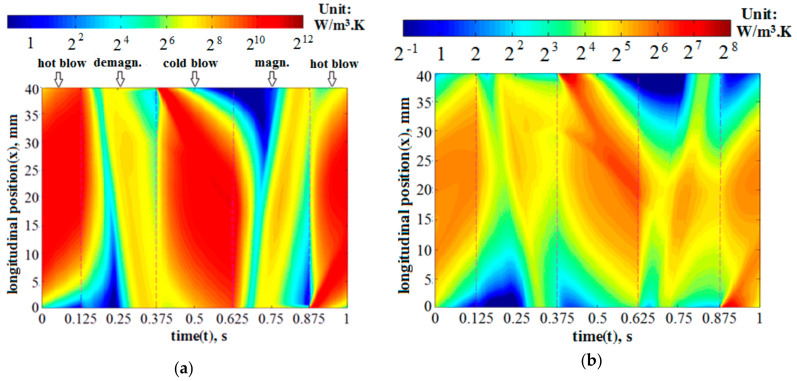
Contours of local entropy generation rates induced by heat transfer, taking the log values based on 2, for the cases with the fluid displacement equal to 0.25, where an entire cycling period is depicted. (**a**) Local fluid entropy generation rate; and (**b**) local solid entropy generation rate.

The variations of specific entropy generation rates of the fluid heat transfer and the total AMR, expressed by $S_{g,\Delta T}^{f}$ and $S_{g}^{\mathrm{AMR}}$, respectively, with relative fluid displacement (φ) are depicted by the left vertical axes in Figure 3. Two thicknesses of the Gd plate (δ_p_ = 0.4 and 0.8 mm) were adopted and Figure 3a–c shows the cycling periods (τ) of 0.5, 1 and 4 s, respectively. It is clear from Figure 3a–c that the $S_{g,\Delta T}^{f}$ and $S_{g}^{\mathrm{AMR}}$ increased with the decreasing τ or rising φ. For example, for the AMR with δ_p_ = 0.4 mm, the φ increased from 0.15 to 0.25, and the $S_{g,\Delta T}^{f}$ and $S_{g}^{\mathrm{AMR}}$ at the τ of 1s varied from 31.5 and 38.4 to 55.5 and 71.2 W/(m^3^·K), respectively. It is evident that these increments were related to the heat transfer enhancement due to a larger φ. Specifically, a larger φ could result in a larger heat transfer rate between the fluid and the solid and, thus, a greater entropy generation of the fluid heat transfer. Moreover, with the increment of φ, the irreversible loss of fresh water and the remanent fluid mixing in the channel may cause the viscous entropy generation rate to increase considerably. As for the solid Gd plates, the entropy generation rate and its increment with rising φ could be limited because it has a larger thermal conductivity. To explore the above phenomenon more clearly, the local mean entropy generation rates of the fluid and solid heat transfer (${\bar{s}}_{g,\Delta T}^{f}$ and ${\bar{s}}_{g,\Delta T}^{s}$) were calculated by averaging the local entropy generation rates over their cross sections, and their variations over an entire cycle (τ = 1 s) are presented in Figure 4a,b and Figure 5a,b for the cases with the φ of 0.15 and 0.25, respectively. The log mean entropy generation rates based on two are adopted in Figure 4 and Figure 5 for better distinction. 

By scrutinizing the subfigures in Figure 4 or Figure 5, it is clearly seen that the fluid ${\bar{s}}_{g,\Delta T}^{f}$ was much larger than the solid ${\bar{s}}_{g,\Delta T}^{s}$. Moreover, during the hot or cold blows, both ${\bar{s}}_{g,\Delta T}^{f}$ and ${\bar{s}}_{g,\Delta T}^{s}$ considerably increased with the increment of fluid displacement (φ). Contrastingly, as the fluid stayed stationary during the magnetization and demagnetization, the ${\bar{s}}_{g,\Delta T}^{f}$ and ${\bar{s}}_{g,\Delta T}^{s}$ in those processes were found to have no significant dependence on φ. All the contours are consistent with the curves of entropy generation rates in Figure 3.

#### 4.3.2. Variation of the Entropy Generation Number

The variations of the entropy generation number (Ns), i.e., the total entropy generation rate normalized by the refrigeration capacity, with fluid displacement (φ) under the conditions with cycling periods (τ) of 0.5, 1 and 4 s are presented in Figure 3a–c, respectively, with the right vertical axes. It is clear from the right axes of Figure 3 that the Ns varied concavely with the increment of φ for all the τ values, and that thinner Gd plates facilitated a smaller pit Ns. Moreover, the smallest pit Ns were obtained by adopting the largest τ. Specifically, for the AMR manufactured by 0.4mm thick Gd plates, when the τ was 0.5, 1 and 4 s, the generated pit Ns were 9.1 × 10^−4^, 4.7 × 10^−4^ and 2.4 × 10^−4^ K^−1^, respectively, and the corresponding optimal φ was 0.1, 0.15 and 0.3.

It is noted that with the increment of φ, the q_ref_ and COP varied convexly, while the Ns changed concavely. Furthermore, the optimal φ for the largest q_ref_ and COP, and the lowest Ns were inconsistent, and were 0.2, 0.4 and 0.3, respectively, in the current work. Thus, some compromises need be made in the optimization of AMR.

## 5. Conclusions

In the current work, a 2D numerical model of stationary grids was developed and numerical simulation was conducted for the reciprocating magnetic refrigerators with Gd plates under the conditions of various structural and operational parameters. By simulation, the effects of the relative fluid displacement (φ) on the specific refrigeration capacity (q_ref_) and the Coefficient of Performance (COP) were obtained, and the variations of the entropy generation rate (S_g_) and number (Ns) were researched. Furthermore, the contours of the local entropy generation rate were presented for discussion. From the current work, some conclusions were obtained:

(1) The q_ref_ and COP of the magnetic refrigerator followed the convex variation trend with increasing φ, and a smaller plate thickness (δ_p_) facilitated a larger peak of the q_ref_ and COP. Furthermore, the largest τ produced the largest COP (=9.11), while the greatest q_ref_ (=151.2 kW/m^3^) was generated at a moderate τ.
(2) With increments of φ, the S_g_ in the AMR rose monotonically, while the Ns varied concavely. Moreover, a larger τ, or a smaller δ_p,_ resulted in a smaller pit Ns, and the lowest Ns was 2.4 × 10^−4^ K^−1^.
(3) The optimal φ for the largest q_ref_ and COP and the lowest Ns was inconsistent, and were 0.2, 0.4 and 0.3, respectively, in the current work. Thus, some compromises need be made in the optimization of AMR.

## References

[1] Russek S.L., Zimm C.B. (2006). Potential for cost effective magnetocaloric air conditioning systems. Int. J. Refrig..

[2] Kitanovski A., Tušek J., Tomc U., Plaznik U., Ozbolt M., Poredoš A. (2015). Magnetocaloric Energy Conversion: From Theory to Applications.

[3] Aprea C., Greco A., Maiorino A. (2015). GeoThermag: A geothermal magnetic refrigerator. Int. J. Refrig..

[4] Tegus O., Bruck E., Buschow K.H., de Boer F.R. (2002). Transition metal-based magnetic refrigerants for room temperature applications. Nature.

[5] Gschneidner K.A., Pecharsky V.K., Tsokol A.O. (2005). Recent developments in magnetocaloric materials. Rep. Prog. Phys..

[6] Bjørk R., Bahl C.R.H., Smith A., Pryds N. (2010). Review and comparison of magnet designs for magnetic refrigeration. Int. J. Refrig..

[7] You Y.H., Guo Y., Xiao S.F., Yu S., Ji H., Luo X. (2016). Numerical simulation and performance improvement of a multi-polar concentric Halbach cylindrical magnet for magnetic refrigeration. J. Magn. Magn. Mater..

[8] Eriksen D., Engelbrecht K., Bahl C.R.H., Bjørk R., Nielsen K.K., Insinga A.R., Pryds N. (2015). Design and experimental tests of a rotary active magnetic regenerator prototype. Int. J. Refrig..

[9] Gomez J.R., Garcia R.F., Catoira A.D.M., Gómez R.M. (2013). Magnetocaloric effect: A review of the thermodynamic cycles in magnetic refrigeration. Renew. Sustain. Energy Rev..

[10] Plaznik U., Tusek J., Kitanovski A., Poredoš A. (2013). Numerical and experimental analyses of different magnetic thermodynamic cycles with an active magnetic regenerator. Appl. Therm. Eng..

[11] You Y.H., Yu S., Tian Y.Q., Luo X., Huang S. (2016). A numerical study on the unsteady heat transfer in active regenerator with multi-layer refrigerants of rotary magnetic refrigerator near room temperature. Int. J. Refrig..

[12] You Y.H., Wu Z.D., Xiao S.F., Li H., Xu X. (2017). A comprehensive two-dimensional numerical study on unsteady conjugate heat transfer in magnetic refrigerator with Gd plates. Int. J. Refrig..

[13] You Y.H., Wu Z.D., Chen P.A., Ji H., Zeng X., Xu X., Dai F. (2018). Improving magnetic refrigerator performances by enhancing convection heat transfer with staggered twin-wedged elements. Appl. Therm. Eng..

[14] Vuarnoz D., Kawanami T. (2012). Numerical analysis of a reciprocating active magnetic regenerator made of gadolinium wires. Appl. Therm. Eng..

[15] Chen Z.H., Utaka Y., Tasaki Y. (2014). Measurement and numerical simulation on the heat transfer characteristics of reciprocating flow in microchannels for the application in magnetic refrigeration. Appl. Therm. Eng..

[16] Arnold D.S., Tura A., Ruebsaat-Trott A., Rowe A. (2014). Design improvements of a permanent magnet active magnetic refrigerator. Int. J. Refrig..

[17] Zimm C., Boeder A., Chell J., Sternberg A., Fujita A., Fujieda S., Fukamichi K. (2006). Design and performance of a permanent magnet rotary refrigerator. Int. J. Refrig..

[18] Lozano J.A., Engelbrecht K., Bahl C.R.H., Nielsen K.K., Barbosa J.R., Prata A.T., Pryds N. (2014). Experimental and numerical results of a high frequency rotating active magnetic refrigerator. Int. J. Refrig..

[19] Bahl C.R.H., Petersen T.F., Pryds N., Smith A. (2008). A versatile magnetic refrigeration test device. Rev. Sci. Instrum..

[20] Tomc U., Tušek J., Kitanovski A., Poredoš A. (2014). A numerical comparison of a parallel-plate AMR and a magnetocaloric device with embodied micro thermoelectric thermal diodes. Int. J. Refrig..

[21] Qian S.X., Yuan L.F., Yu J.L., Yan G. (2018). Variable load control strategy for room-temperature magnetocaloric cooling applications. Energy.

[22] Aprea C., Greco A., Maiorino A. (2017). An application of the artificial neural network to optimize the energy performances of a magnetic refrigerator. Int. J. Refrig..

[23] Gschneidner K.A., Pecharsky V.K. (2008). Review: Thirty years of near room temperature magnetic cooling: Where we are today and future prospects. Int. J. Refrig..

[24] Yu B., Liu M., Egolf P.W., Kitanovski A. (2010). A review of magnetic refrigerator and heat pump prototypes built before the year 2010. Int. J. Refrig..

[25] Aprea C., Maiorino A. (2010). A flexible numerical model to study an active magnetic refrigerator for near room temperature applications. Appl. Energy.

[26] Nielsen K.K., Bahl C.R.H., Smith A., Bjørk R., Pryds N., Hattel J. (2009). Detailed numerical modeling of a linear parallel-plate Active Magnetic Regenerator. Int. J. Refrig..

[27] Nielsen K.K., Tušek J., Engelbrecht K., Schopfer S., Kitanovski A., Bahl C.R.H., Smith A., Pryds N., Poredos A. (2011). Review on numerical modeling of active magnetic regenerators for room temperature applications. Int. J. Refrig..

[28] Tušek J., Kitanovski A., Poredoš A. (2013). Geometrical optimization of packed-bed and parallel-plate active magnetic regenerators. Int. J. Refrig..

[29] Kamran M.S., Ali H., Farhan M., Tang Y.B., Chen Y.G., Wang H.S. (2016). Performance optimization of room temperature magnetic refrigerator with layered/multi-material microchannel regenerators. Int. J. Refrig..

[30] Monfared B. (2018). Design and optimization of regenerators of a rotary magnetic refrigeration device using a detailed simulation model. Int. J. Refrig..

[31] Teyber R., Trevizoli P.V., Christiaanse T.V., Govindappa P., Niknia I., Rowe A. (2018). Semi-analytic AMR element model. Appl. Therm. Eng..

[32] Bejan A. (1996). Method of entropy generation minimization, or modeling and optimization based on combined heat transfer and thermodynamics. Rev. Gen. Therm..

[33] Cheng X.T., Liang X.G. (2013). Discussion on the applicability of entropy generation minimization to the analyses and optimizations of thermodynamic processes. Energy Convers. Manag..

[34] Turkakar G., Ozyurt T.O. (2015). Entropy generation analysis and dimensional optimization of an evaporator for use in a microscale refrigeration cycle. Int. J. Refrig..

[35] You Y.H., Fan A.W., Liang Y.M., Jin S., Liu W., Daia F. (2015). Entropy generation analysis for laminar thermal augmentation with conical strip inserts in horizontal circular tubes. Int. J. Therm. Sci..

[36] Rowe A.M., Barclay J.A. (2003). Ideal magnetocaloric effect for active magnetic regenerators. J. Appl. Phys..

[37] Li P., Gong M., Wu J. (2008). Geometric optimization of an active magnetic regenerative refrigerator via second-law analysis. J. Appl. Phys..

[38] Lei T., Engelbrecht K., Nielsen K.K., Veje C.T. (2017). Study of geometries of active magnetic regenerators for room temperature magnetocaloric refrigeration. Appl. Therm. Eng..

[39] Trevizoli P.V., Alcalde D.P., Barbosa J.R. Second law optimization of regenerative geometries for magnetic cooling applications. Proceedings of the 6th International Conference on Magnetic Refrigeration.

[40] Morrish A.H. (1965). The Physical Principles of Magnetism.

[41] Petersen T.F., Pryds N., Smith A., Hattel J., Schmidt H., Knudsen H.H. (2008). Two-dimensional mathematical model of a reciprocating room-temperature Active Magnetic Regenerator. Int. J. Refrig..

